# Unibody Endograft Using AFX 2 for Less Invasive and Faster Endovascular Aortic Repair: Protocol for a Multicenter Nonrandomized Study

**DOI:** 10.2196/16959

**Published:** 2020-04-06

**Authors:** Roberto Silingardi, Pasqualino Sirignano, Francesco Andreoli, Wassim Mansour, Mattia Migliari, Francesco Speziale, LIVE Study Collaborators

**Affiliations:** 1 Department of Vascular Surgery Ospedale Civile Sant'Agostino-Estense, Azienda Ospedaliero-Universitaria di Modena University of Modena and Reggio Emilia Modena Italy; 2 Vascular and Endovascular Surgery Unit Department of Surgery Paride Stefanini Sapienza University of Rome Rome Italy; 3 See Acknowledgments section for collaborators/group members

**Keywords:** aortic aneurysm, abdominal aortic aneurysm, endovascular aortic repair, endovascular repair, AFX 2, long-term results

## Abstract

**Background:**

Since the introduction of endovascular aortic repair (EVAR) for treatment of abdominal aortic aneurysms (AAAs), progressive improvements in results have been achieved. However, conventional bifurcated stent grafts have been proven to have a nonnegligible risk of failure and secondary intervention, principally due to the lack of adequate proximal sealing. The unique AFX 2 Endovascular AAA System (Endologix, Irvine, CA) unibody device, which provides different sealing and fixation features compared with conventional devices, seems to overcome these limitations.

**Objective:**

The aim of this study is to evaluate intraoperative, perioperative, and postoperative results in patients treated with the AFX 2 Endovascular AAA System endografts for elective AAA repair in a large cohort of consecutive patients.

**Methods:**

All eligible EVAR patients will be included in this observational, multicenter, prospective, nonrandomized study. The number of patients to be enrolled is 500.

**Results:**

The primary endpoint of the study is to evaluate the technical and clinical success of EVAR with unibody endografts in short- (90-day), mid- (1-year), and long-term (5-year) follow-up periods. The following secondary endpoints will also be addressed: operative time, intraoperative radiation exposure, contrast medium usage, AAA sac shrinkage at 12-month and 5-year follow-up, and any potential role of patients’ baseline characteristics and device configuration on primary endpoint. The actual start date of the investigation was November 2019. The final patient is expected to be treated by the end of December 2020, and the estimated study completion date is December 2025.

**Conclusions:**

This study will provide verified real-world data on AAAs treated by AFX 2 endografts and followed for a long-term interval.

**International Registered Report Identifier (IRRID):**

PRR1-10.2196/16959

## Introduction

In recent years, endovascular aortic repair (EVAR) has emerged as a safe and valid option for treatment of abdominal aortic aneurysms (AAAs) (Multimedia Appendix 1). The AFX 2 Endovascular AAA System (Endologix, Irvine, CA) consists of two components: an implantable stent graft and a disposable delivery catheter. The preloaded stent graft is transferred through the AFX 2 Introducer System and inserted endoluminally via the femoral or iliac artery over a guidewire and, upon deployment and withdrawal of the delivery system, expands to the indicated diameter. During deployment and expansion, the stent graft is intended to form proximal and distal seal zones surrounding the aneurysm location. The stent graft is composed of a cobalt-chromium alloy self-expanding stent cage with a thin-walled, low porosity expanded polytetrafluoroethylene graft cover that is attached proximally and distally to the stent cage with a polypropylene suture. The system consists of a unibody bifurcated stent graft, with proximal extension and limb extension accessory components available, as needed, to accommodate the patient’s specific anatomy.

The AFX 2 Endovascular AAA System is essentially composed of two distinct elements: the bifurcated stent graft and the proximal and iliac limb extension stent grafts.

The bifurcated element is the primary component that is delivered into the patient’s aorta. All bifurcated stent grafts consist of a unibody configuration (an aortic main body with two attached iliac legs). The main body and each iliac leg are constructed from a single wire. The main body is manufactured in sizes ranging from 22 mm to 28 mm. The iliac legs are 13 mm to 20 mm in diameter for all sizes of bifurcated stent grafts.

The proximal and iliac limb extension stent graft components are used to extend the lengths of implanted bifurcated components. The AFX 2 Endovascular AAA System proximal extensions (Vela) are available in suprarenal and infrarenal configurations and use a circumferential radiopaque marker for identification of the proximal graft fabric line.

According to current instructions for use, the following anatomical criteria are required.

- Adequate iliac or femoral access compatible with the required delivery systems (diameter 6.5 mm)
- Nonaneurysmal aortic neck between the renal arteries and the aneurysm with a length 15 mm, a diameter ≥18 mm and ≤32 mm, and a neck angle ≤60° to the body of the aneurysm
- Aortic length ≥1.0 cm longer than the body portion of the chosen bifurcated model
- Common iliac artery distal fixation site with a distal fixation length ≥15 mm, ability to preserve at least one hypogastric artery, a diameter ≥10 mm and ≤23 mm, and an iliac angle ≤90° to the aortic bifurcation
- Extension stent grafts must have the ability to overlap the bifurcated stent graft by at least 30 to 40 mm proximally and at least 15 to 20 mm distally

Some elegant papers have been published on this unique device [1-7]. In 2010, Carpenter et al [6] reported in a study of 157 patients treated by unibody device implantation in three different prospective multicenter trials (Powerlink trial, Powerlink XL trial, and Powerlink Suprarenal Extension trial). All enrolled patients were treated between 2000 and 2008 and followed through 5 years. Technical success was achieved in 99% of patients. Aneurysm exclusion was achieved in all patients over a mean procedure time of 132 (SD 58) minutes. No aneurysm related deaths, ruptures, conversions, or migrations have been observed to current follow-up, as these aneurysms have continued to remodel with more than 92% of patients free of sac growth. At each annual evaluation period, no stent fractures, material failures, or losses of patency were found by the core laboratory. During the follow-up period, 5 patients were treated for a Type Ia endoleak, 3 for a Type Ib, and 3 for a limb occlusion. All of these reinterventions were performed with a new endovascular procedure without needing surgical conversion [6].

Similar results were reported by Qu and Raithel [4] in their single center study on more than 600 patients. Among the 612 patients in the cohort, 99 cases (16%) completed between 1999 and 2004, had the endograft deployed from the renal artery downward. The remaining 513 (84%) had the bifurcated stent graft deployed onto the native bifurcation, and among those cases 146 (28%) were deemed as challenging anatomy with a short or angulated neck. Technical success was achieved in 98.5% of patients (603/612). Intraoperative conversion occurred in 9 patients: 8 delivery access failures and 1 rupture. Perioperatively, 3 deaths occurred, and 2 limb occlusions were encountered. The rates of late conversion in the renal fixation and anatomical fixation groups were 4.0% and 1.9%, respectively. Likewise, the cumulative rates of a type I proximal endoleak in the renal fixation and anatomical fixation groups were 5.0% and 1.2%, respectively. Remarkably, no stent fracture, graft disruption, or type III or type IV endoleak was observed in their experience. Freedom from aneurysm sac diameter increase was 96% [4].

Moreover, Silingardi et al [7] performed a comparative study to compare nephrotoxic contrast medium with radiation exposure during elective EVAR procedures using unibody and modular devices. The initial hypothesis was that the unique unibody device structure, associated with not needing a gate cannulation, could reduce total procedural and total fluoroscopy time, as well as reduce the volume of contrast medium needed. Their study on 60 unibody devices and 57 bifurcated devices confirmed the hypothesis. For unibody and bifurcated devices, the median surgical procedure duration was 75 min vs 105 min (*P*<.001), the median volume of iodine contrast injected was 85 ml vs 170 ml (*P*<.001), and the median fluoroscopy time was 350 sec vs 780 sec (*P*<.001), respectively [7].

The promising data from these studies should be confirmed by prospective data collecting in a large consecutive cohort of patients using the latest generation unibody device implantation. Therefore, this study aims to evaluate intraoperative, perioperative, and postoperative results in patients treated with AFX 2 Endovascular AAA System endografts for elective AAA repair in a large cohort of consecutive patients.

## Methods

### Objective and Duration of Investigation

The aim of this study is to evaluate intraoperative, perioperative, and postoperative results in patients treated using the latest generation AFX 2 Endovascular AAA System endograft for elective AAA repair in a multicentric study.

A total of 46 south European high-volume centers across Italy and Spain were involved in the Less Invasive and Faster Endovascular Aortic Repair Study. In a 12-month period from October 2018 to October 2019, mean EVAR procedures per center were 49.56 (range 20-140), while mean AFX procedures per year per center were 14.54 (range 10-57).

All consecutive eligible patients submitted to EVAR by AFX 2 Endovascular AAA System implantation will be included in the analysis. Patients will be submitted to EVAR procedures on the basis of their own preferences, anatomical features, and the operator’s experience.

In light of the participating centers’ numbers and activity volumes, an estimated 500 patients submitted to EVAR with AFX 2 should be enrolled. The sample size is low enough to obtain statistically significative results, according to previous published studies [1-7]. The anticipated duration of this clinical investigation is approximately 6 years. It is estimated that the inclusion period will be 12 months. The follow-up period is set to be 5 years.

Prior to enrollment in the clinical investigation, patients will be evaluated by their physician for the inclusion criteria. Each patient’s medical condition should be stable, with no underlying medical condition that would prevent them from performing the required testing or completing the study. Patients should be geographically stable, willing and able to cooperate in this clinical study, and remain available for a midterm follow-up. Patients who do not wish to participate in this study can obtain the best available EVAR therapy as indicated, that is refusal to participate in this study will in no way affect their care at the institution. Inclusion and exclusion criteria are detailed in Textbox 1.

**Inclusion and exclusion criteria for the study.**

**Inclusion criteria**

- Elective abdominal aortic aneurysm patients that should be treated by standard abdominal endovascular aneurysm repair, according to Endologix AFX unibody device’s instructions for use
- Patient is willing to comply with specified follow-up evaluations at the specified times for the duration of the study
- Patient is >18 years of age
- Patient, or their legal representative, understands the nature of the procedure and provides written informed consent prior to enrollment in the study

**Exclusion criteria**

- Abdominal endovascular aneurysm repair performed in urgent or emergent setting
- Patients treated outside Endologix AFX unibody device’s instructions for use
- Patients refusing treatment
- Patients for whom antiplatelet therapy, anticoagulants, or antihypertensive drugs are contraindicated
- Patients with a history of prior life-threatening contrast medium reaction
- Life expectancy is less than follow-up period

This study respects all the principles reported in the current version of the Helsinki declaration (2013). According to the current International Council for Harmonisation of Technical Requirements for Pharmaceuticals for Human Use and Good Clinical Practice guidelines, each investigator is responsible for the regularity of the study. The aim of these standards is to assure the safety and comfort of all people recruited in the study. The study protocol and the written informed consensus form will be submitted to the local ethics committees for review.

AAA morphology will be assessed by OsiriX MD (PIXMEO, Geneva, Switzerland) on a regular Mac OS computer in one preoperative, contrast-enhanced, computed tomography angiography (CTA) [8]. The CTA must be performed with a biphasic acquisition protocol (unenhanced and contrast-enhanced scanning with a bolus tracking system) and reconstructions of 1-mm slices. All measurements (diameter, length, and angle) will be evaluated on a workstation with dedicated reconstruction software for center lumen line analysis and multiplanar reconstruction.

A patient is considered enrolled in the study if there is full compliance with the study inclusion and exclusion criteria.

Clinical data will be collected at patient enrollment, the EVAR procedure, discharge, planned follow-ups (ie, 1-3 months and 12 months postprocedure, and yearly thereafter), unplanned or interim follow-ups, and patient death. CTAs are mandatory within 90 days and then at 1 and 5 years after the index procedure. The duplex ultrasound scan will be performed at the same follow-up interval and also at 24, 36, and 48 months. A new CTA will be performed in case of unexpected events during follow-up.

### Endpoints

The primary endpoint of the study is to evaluate the technical and clinical success of EVAR with unibody endografts in short- (90-day), mid- (1-year), and long-term (5-year) follow-up periods.

Technical success was defined as the correct graft deployment without any unintentional occlusion of the aortic visceral branches or both hypogastric arteries, with aneurysm exclusion confirmed by the intraoperative angiography, no signs of type I or III endoleak, or conversion to open surgery.

Clinical success included successful deployment of the endovascular device at the intended location without death as a result of aneurysm-related treatment, type I or III endoleak, graft infection or thrombosis, aneurysm expansion (>5 mm), aneurysm rupture, or conversion to open repair (OR), as well as the presence of graft dilatation of 20% or more by diameter, graft migration, or a failure of device integrity [9].

The clinical and technical success were defined as assisted in cases of unplanned endovascular procedures, or secondary if unplanned surgery is necessary.

The following secondary endpoints will be also addressed: operative time, intraoperative radiation exposure, contrast medium usage, AAA sac shrinkage at 12-month and 5-year follow-ups, and any potential role of patients’ baseline characteristics and device configuration on primary endpoint.

### Data Collection and Analysis

Patient data will be captured electronically using a computer-based platform accessible to all investigators. Descriptive data summaries will be used to present and summarize the collected data. For categorical variables such as gender, frequency distributions and cross tabulations will be given. For numeric variables such as patient age, minimum, maximum, mean, median, and standard deviation will be calculated. For all variables, a 95% confidence interval for the relevant parameters of the underlying distribution will be calculated. For all time-dependent events, life tables will be calculated using the Kaplan-Meier estimate method for a period starting on the date of the procedure up to and including all follow-up visits. Stratification to risk factors will be performed and the logrank test will be used to compare the different outcomes; associated *P*<.05 will be defined as significant.

All preoperative and follow-up CTAs were assessed and independently evaluated by two experienced vascular surgeons at core lab centers. Disagreements will be discussed and resolved by consensus.

### Patient Confidentiality

All information and data concerning patients or their participation in this clinical investigation will be considered confidential. Only authorized personnel will have access to these confidential files. Authorized personnel of health authorities will have the right to inspect and copy all records pertinent to this clinical investigation. All data used in the analysis and reporting of this clinical investigation will be anonymized.

## Results

The actual start date of the investigation was November 2019. It is anticipated that 500 patients will be recruited to the study. The final patient is expected to be treated by the end of December 2020 and the estimated study completion date is December 2025. After data analysis, results will be shared with each investigator.

## Discussion

In the last years, EVAR has become the standard of care for AAA treatment, and nowadays it represents the recommended modality of treatment according to the European Society for Vascular and Endovascular Surgery and the Society for Vascular Surgery guidelines [10,11].

However, the major randomized controlled trials (RCTs) on EVAR vs OR have not reached definitive conclusions. In 2004, the results of the first two RCTs were published [12,13].

The EVAR-1 trial described a clear advantage of EVAR compared to OR at 30 days. Greenhalgh and collaborators [12] reported that 30-day mortality in the EVAR group was 1.7% (9/531) vs 4.7% (24/516) in the OR group (*P*=.009). Their results were interpreted as a license to continue scientific evaluation of EVAR, but not to change clinical practice [12].

More enthusiastic conclusions came from the analysis of the Dutch Randomised Endovascular Aneurysm Management (DREAM) trial, reporting an operative mortality rate of 4.6% in the OR group (8/174 patients) and 1.2% in the EVAR group (2/171 patients) in a series of patients treated between 2000 and 2003, resulting in a risk ratio of 3.9 (95% CI 0.9-32.9). The combined rate of operative mortality and severe complications was 9.8% in the OR group (17/174 patients) and 4.7% in the EVAR group (8/171 patients), resulting in a risk ratio of 2.1 (95% CI 0.9-5.4). The authors concluded that EVAR was preferable to OR [13].

However, long-term follow-up was demanded to determine whether advantages persisted, and 1 year later, both trials published their midterm results [14,15]. In the EVAR-1 trial, all-cause mortality was similar in the two groups (approximately 28%; *P*=.46), but a persistent reduction in AAA-related deaths was recorded in the EVAR group compared with the OR group (4% vs 7%; *P*=.04). On the other hand, the proportion of patients with postoperative complications was 41% in the EVAR group and 9% in the OR group (*P*<.001) [14]. In the DREAM trial, 2 years after randomization, the cumulative survival rates were 89.6% for OR and 89.7% for EVAR and the cumulative rates of AAA-related deaths were 5.7% for OR and 2.1% for EVAR. This advantage of EVAR over OR was accounted for by events occurring in the perioperative period, with no significant difference in subsequent AAA-related mortality. The rate of survival free of moderate or severe complications was also similar in the two groups at 2 years (65.9% OR vs 65.6% EVAR) [15].

The Open Versus Endovascular Repair (OVER) trial, which included patients treated between 2002 and 2007, was published in 2010. On the basis of a mean follow-up of 1.8 years, the OVER trial results showed that perioperative mortality was lower for EVAR than for OR (0.5% vs 3.0%; *P*=.004), without any difference at 2 years (7.0% vs 9.8%, *P*=.13). Mortality after the perioperative period was similar in the two groups (6.1% vs 6.6%); however, 4 late deaths in the EVAR group were AAA-related compared with none in the OR group. No differences between the two groups in terms of major morbidity, procedure failure, secondary intervention, AAA-related hospitalization, or health-related quality of life were recorded. Interestingly, no increase in midterm mortality after EVAR resulted in the loss of early survival advantage as shown in previous trials was observed [16].

A year later, a French RCT (ACE Trial) reported quite different results with no differences between EVAR and OR. Although only low to intermediate risk patients were enrolled, OR and EVAR offered no difference in survival (96.5% vs 95.2% at 1 year, and 86.7% vs 86.3% at 3 years) or in major and minor complications (95.9% vs 93.2% at 1 year, and 85.1% vs 82.4% at 3 years) [17].

These results led to a change in point of view: EVAR was considered as feasible as OR without any advantages, even in the short-term. The same year, a new US study, with a 6-year follow-up on 45,652 Medicare beneficiaries undergoing EVAR or OR in the period between 2001 and 2004, was analyzed to clarify the late results of endovascular procedures. Throughout follow-up, overall reintervention or readmission rates were similar with the two repair methods but slightly more common after EVAR than OR (7.6 vs 7.0/100 person-years; *P*<.001). Overall 30-day mortality with any reintervention or readmission was 9.1%. EVAR patients had more AAA-related reinterventions than OR (3.7% vs 0.9%; *P*<.001; mortality 5.6%). Conversely, EVAR patients had fewer laparotomy-related reinterventions than OR patients (1.4% vs 3.0%; *P*<.001; mortality, 8.1%) and fewer readmissions without surgery (2.0% vs 2.7%; *P*<.001; mortality 10.9%). Overall, reinterventions and readmissions accounted for 9.6% of all EVAR deaths and 7.6% of all OR deaths in the follow-up period (*P*<.001). The authors concluded that reintervention and readmission were slightly higher after EVAR. Survival was negatively affected by reintervention or readmission after EVAR and OR [18].

In 2016, the long-term results of the EVAR-1 RCT were published. In a mean of 12.7 years for follow-up, Patel et al [19] reported a similar overall mortality in the EVAR and OR groups (9.3 deaths per 100 person-years vs 8.9 deaths per 100 person-years; hazard ratio (HR) 1.11, 95% CI 0.97-1.27). However, a time analysis showed that beyond 8 years after randomization OR had a significantly lower mortality (HR 1.25, 95% CI 1.00-1.56, for total mortality; and HR 5.82, 95% CI 1.64-20.65, *P*=.006 for aneurysm-related mortality) mainly due to secondary sac rupture. The authors concluded that the early benefits of EVAR in terms of mortality were lost in the long-term [19].

However, late results from the OVER trial were published. In Lederle’s [20] study, long-term overall survival was similar between patients who underwent endovascular repair and those who underwent OR (EVAR 68% vs OR 70%; HR 0.96, 95% CI 0.82-1.13). A difference between groups was noted in the number of patients who underwent secondary therapeutic procedures (EVAR 26.7% vs OR 19.8; 95% CI 2.0-17.5). Their results were not consistent with the findings of worse performance of endovascular repair with respect to long-term survival that was seen in the two European trials [20].

Notably, all these trials reported only few data or none at all on patients treated by unibody stent graft implantation. As mentioned above, AFX 2 Endovascular AAA System endografts are completely different from a technical and philosophical point of view from all other modular devices, and it seems to also provide different results. In fact, different studies have already demonstrated the advantages of this endograft and its safety and efficacy in short and midterm follow-up periods [1-5]. The preservation of the aortic bifurcation focuses the disrupting forces caused by columnar blood on the carrefour rather than on the aortic neck minimizing migration risk [1,2]. The obviation of contralateral gate cannulation makes this graft faster to deploy and uniquely suitable in cases of narrow aortic bifurcations. Moreover, this graft is allowed for future contralateral access for lower extremity interventions in a patient population with different vascular diseases. Finally, Dietrich et al [5] affirmed that the big contact area provided by the fabric free to move with the blood pressure wave can promote sac shrinkage and contrast type II endoleak formations.

Given the lack in current literature of effective data on unibody endograft results, the aim of our prospective study is addressing intraoperative, perioperative, and postoperative results in patients treated by the latest generation AFX 2 Endovascular AAA System endografts for elective AAA repair in a multicentric study.

## Appendix

Multimedia Appendix 1 EVAR Overview.

## Abbreviations

AAA: abdominal aortic aneurysm
CTA: computed tomography angiography
DREAM: Dutch Randomized Endovascular Aneurysm Management
EVAR: endovascular aortic repair
HR: hazard ratio
OR: open repair
OVER: Open Versus Endovascular Repair
RCT: randomized controlled trial

## References

[1] Coppi G, Silingardi R, Tasselli S, Gennai S, Saitta G, Veraldi GF (2008). Endovascular treatment of abdominal aortic aneurysms with the Powerlink Endograft System: influence of placement on the bifurcation and use of a proximal extension on early and late outcomes. J Vasc Surg.

[2] Wang GJ, Carpenter JP, Endologix Investigators (2008). The Powerlink system for endovascular abdominal aortic aneurysm repair: six-year results. J Vasc Surg.

[3] Albertini J, Lahlou Z, Magnan P, Branchereau A, French Powerlink Multicenter Trial Investigators (2005). Endovascular repair of abdominal aortic aneurysms with a unibody stent-graft: 3-year results of the French Powerlink Multicenter Trial. J Endovasc Ther.

[4] Qu L, Raithel D (2009). From clinical trials to clinical practice: 612 cases treated with the Powerlink stent-graft for endovascular repair of AAA. J Cardiovasc Surg (Torino).

[5] Diethrich EB (2014). Novel sealing concept in the Endologix AFX unibody stent-graft. J Cardiovasc Surg (Torino).

[6] Carpenter JP, Garcia MJ, Harlin SA, Jordan WD, Jung MT, Krajcer Z, Rodriguez-Lopez JA (2010). Contemporary results of endovascular repair of abdominal aortic aneurysms: effect of anatomical fixation on outcomes. J Endovasc Ther.

[7] Silingardi R, Azzoni I, Giuliani E, Saitta G, Gennai S, Coppi G (2015). Unibody endografts for abdominal aortic aneurysm repair reduce radiation and nephrotoxic exposure compared with modular endografts. Ann Vasc Surg.

[8] Setacci F, Sirignano P, Cappelli A, Setacci C (2012). The wonders of a newly available post-analysis CT software in the hands of vascular surgeons. Eur J Vasc Endovasc Surg.

[9] Chaikof EL, Blankensteijn JD, Harris PL, White GH, Zarins CK, Bernhard VM, Matsumura JS, May J, Veith FJ, Fillinger MF, Rutherford RB, Kent KC, Ad Hoc Committee for Standardized Reporting Practices in Vascular Surgery of The Society for Vascular Surgery/American Association for Vascular Surgery (2002). Reporting standards for endovascular aortic aneurysm repair. J Vasc Surg.

[10] Wanhainen A, Verzini F, Van Herzeele I, Allaire E, Bown M, Cohnert T, Dick F, van Herwaarden J, Karkos C, Koelemay M, Kölbel T, Loftus I, Mani K, Melissano G, Powell J, Szeberin Z, de Borst GJ, Chakfe N, Debus S, Hinchliffe R, Kakkos S, Koncar I, Kolh P, Lindholt JS, de Vega M, Vermassen F, Björck M, Cheng S, Dalman R, Davidovic L, Donas K, Earnshaw J, Eckstein H-H, Golledge J, Haulon S, Mastracci T, Naylor R, Ricco J-B, Verhagen H, Esvs Guidelines Committee (2019). Editor's choice - European Society for Vascular Surgery (ESVS) 2019 clinical practice guidelines on the management of abdominal aorto-iliac artery aneurysms. Eur J Vasc Endovasc Surg.

[11] Chaikof EL, Dalman RL, Eskandari MK, Jackson BM, Lee WA, Mansour MA, Mastracci TM, Mell M, Murad MH, Nguyen LL, Oderich GS, Patel MS, Schermerhorn ML, Starnes BW (2018). The Society for Vascular Surgery practice guidelines on the care of patients with an abdominal aortic aneurysm. J Vasc Surg.

[12] Greenhalgh RM, Brown LC, Kwong GPS, Powell JT, Thompson SG, EVAR trial participants (2004). Comparison of endovascular aneurysm repair with open repair in patients with abdominal aortic aneurysm (EVAR trial 1), 30-day operative mortality results: randomised controlled trial. Lancet.

[13] Prinssen M, Verhoeven ELG, Buth J, Cuypers PWM, van Sambeek MRHM, Balm R, Buskens E, Grobbee DE, Blankensteijn JD, Dutch Randomized Endovascular Aneurysm Management (DREAM) Trial Group (2004). A randomized trial comparing conventional and endovascular repair of abdominal aortic aneurysms. N Engl J Med.

[14] EVAR trial participants (2005). Endovascular aneurysm repair versus open repair in patients with abdominal aortic aneurysm (EVAR trial 1): randomised controlled trial. Lancet.

[15] Blankensteijn JD, de Jong SECA, Prinssen M, van der Ham AC, Buth J, van Sterkenburg SMM, Verhagen HJM, Buskens E, Grobbee DE, Dutch Randomized Endovascular Aneurysm Management (DREAM) Trial Group (2005). Two-year outcomes after conventional or endovascular repair of abdominal aortic aneurysms. N Engl J Med.

[16] Lederle FA, Freischlag JA, Kyriakides TC, Padberg FT, Matsumura JS, Kohler TR, Lin PH, Jean-Claude JM, Cikrit DF, Swanson KM, Peduzzi PN, Open Versus Endovascular Repair (OVER) Veterans Affairs Cooperative Study Group (2009). Outcomes following endovascular vs open repair of abdominal aortic aneurysm: a randomized trial. JAMA.

[17] Becquemin J, Pillet J, Lescalie F, Sapoval M, Goueffic Y, Lermusiaux P, Steinmetz E, Marzelle J, trialists ACE (2011). A randomized controlled trial of endovascular aneurysm repair versus open surgery for abdominal aortic aneurysms in low- to moderate-risk patients. J Vasc Surg.

[18] Giles KA, Landon BE, Cotterill P, O'Malley AJ, Pomposelli FB, Schermerhorn ML (2011). Thirty-day mortality and late survival with reinterventions and readmissions after open and endovascular aortic aneurysm repair in Medicare beneficiaries. J Vasc Surg.

[19] Patel R, Sweeting MJ, Powell JT, Greenhalgh RM, EVAR Trial Investigators (2016). Endovascular versus open repair of abdominal aortic aneurysm in 15-years' follow-up of the UK endovascular aneurysm repair trial 1 (EVAR trial 1): a randomised controlled trial. Lancet.

[20] Lederle FA, Kyriakides TC, Stroupe KT, Freischlag JA, Padberg FT, Matsumura JS, Huo Z, Johnson GR, OVER Veterans Affairs Cooperative Study Group (2019). Open versus endovascular repair of abdominal aortic aneurysm. N Engl J Med.

