# Correction: Unibody Endograft Using AFX 2 for Less Invasive and Faster Endovascular Aortic Repair: Protocol for a Multicenter Nonrandomized Study

**DOI:** 10.2196/20698

**Published:** 2020-06-24

**Authors:** Roberto Silingardi, Pasqualino Sirignano, Francesco Andreoli, Wassim Mansour, Mattia Migliari, Francesco Speziale, LIVE Study Collaborators

**Affiliations:** 1 Department of Vascular Surgery Ospedale Civile Sant'Agostino-Estense, Azienda Ospedaliero-Universitaria di Modena University of Modena and Reggio Emilia Modena Italy; 2 Vascular and Endovascular Surgery Unit Department of Surgery Paride Stefanini Sapienza University of Rome Rome Italy; 3 See Acknowledgments section for collaborators/group members

In “Unibody Endograft Using AFX 2 for Less Invasive and Faster Endovascular Aortic Repair: Protocol for a Multicenter Nonrandomized Study” (JMIR Res Protoc 2020;9(4):e16959) there were two errors in the Collaborators List.

The collaborator Sonia Ronchey was inadvertently not included in the collaborator list. Additionally, the collaborator name Pietro Volpet should have been listed as Pietro Volpe.

The Collaborator List was initially published as follows:

The LIVE Study Collaborators are as follows: Giancarlo Accarino; Dimitri Apostolou; Guido Bajardi; Stefano Bartoli; Filippo Benedetto; Franco Briolini; Stefano Camparini; Emidio Costantini; Giovanni Credi; Ruggiero Curci; Raffaello Dallatana; Gianmarco de Donato; Carlo Dionisi; Vittorio Dorrucci; Leonardo Ercolini; Gianfranco Fadda; Mauro Ferrari; Loris Flora; Andrea Gaggiano; Roberto Gattuso; Franco Grego; Sabrina Grimaldi; Giovanni Impedovo; Arnaldo Ippoliti; Antonio Jannello; Sergio Losa; Nicola Mangialardi; Isaac Martinez; Javier Martinez; Stefano Michelagnoli; Giancarlo Palasciano; Vincenzo Palazzo; Domenico Palombo; Raffaele Pulli; Giovanni Rossi; Antonino Scolaro; Gianantonio Simoni; Francesco Spinelli; Francesco Talarico; Maurizio Taurino; Marco Trogolo; Nicola Tusini; Gianfranco Veraldi; Pier Francesco Veroux; Gennaro Vigliotti; and Pietro Volpet.

The Collaborator List has now been updated to the following:

The LIVE Study Collaborators are as follows: Giancarlo Accarino; Dimitri Apostolou; Guido Bajardi; Stefano Bartoli; Filippo Benedetto; Franco Briolini; Stefano Camparini; Emidio Costantini; Giovanni Credi; Ruggiero Curci; Raffaello Dallatana; Gianmarco de Donato; Carlo Dionisi; Vittorio Dorrucci; Leonardo Ercolini; Gianfranco Fadda; Mauro Ferrari; Loris Flora; Andrea Gaggiano; Roberto Gattuso; Franco Grego; Sabrina Grimaldi; Giovanni Impedovo; Arnaldo Ippoliti; Antonio Jannello; Sergio Losa; Nicola Mangialardi; Isaac Martinez; Javier Martinez; Stefano Michelagnoli; Giancarlo Palasciano; Vincenzo Palazzo; Domenico Palombo; Raffaele Pulli; Sonia Ronchey; Giovanni Rossi; Antonino Scolaro; Gianantonio Simoni; Francesco Spinelli; Francesco Talarico; Maurizio Taurino; Marco Trogolo; Nicola Tusini; Gianfranco Veraldi; Pier Francesco Veroux; Gennaro Vigliotti; and Pietro Volpe.

The correction will appear in the online version of the paper on the JMIR website on June 24, 2020, together with the publication of this correction notice. Because this was made after submission to PubMed, PubMed Central, and other full-text repositories, the corrected article has also been resubmitted to those repositories.

