# Supplementary material for: Unibody Endograft Using AFX 2 for Less Invasive and Faster Endovascular Aortic Repair: Protocol for a Multicenter Nonrandomized Study
Source: JMIR Res Protoc. 2020 Apr 6;9(4):e16959. doi: 10.2196/16959 (PMC7171559; doi:10.2196/16959)
Supplement: Multimedia Appendix 1 [file resprot_v9i4e16959_app1.docx]

***MULTIMEDIA APPENDIX***

***EVAR OVERVIEW***

Endovascular AAA repair was initially described by Volodos in 1986 [1]. Later, in 1991, Juan Parodi published his experience with retrograde deployment through the femoral arteries of a stent-anchored Dacron prosthetic graft that would act to depressurize the aneurysm sac and thus reduce the risk of aneurysm rupture [2]. From there the field of vascular surgery was radically transformed and the era of EVAR was born. The first generation’s grafts were constructed by suturing a balloon-expandable or self-expanding stent to a Dacron prosthesis. These early grafts were tubular or “aorto-aortic” in design. In 1993, the unibody bifurcated aorto-bisiliac stent was developed [3] and the modular bifurcated design quickly followed [4]. The modern version of endoprosthesis design is a bifurcated graft, commonly using a modular system to allow flexibility with regard to patient anatomy. Currently, several approved infrarenal endografts are commercially available and each device varies in terms of fabric and stent composition, as well as method and anatomic location of fixation. The most popular stent graft designs have favoured suprarenal stents as a means to prevent downward migration and development of type I endoleak, causes of endograft failure. Additionally, some stents have hooks and barbs to provide active, rather than passive, fixation. The conventional modular design elevates the flow divider from the native bifurcation subjecting the graft to downward migration forces. The use of active and passive fixation elements reduces the incidence of migration and consequential EVAR failure, but a careful consideration of the neck is necessary. It is evident that the stability of modular endograft is based only on the infrarenal or suprarenal fixation site, so the neck anatomy, sometimes challenging, and the physiological or pathological neck enlargement are the Achille’s hell of the most of the endograft [5] .

Differently from others commercially available endografts, AFX 2 Endovascular AAA device (Endologix, Inc, Irvine, CA – USA) is based on a fully supported long body design with short legs. This particular design enables to deploy the main body on the aorto-iliac bifurcation providing the so called “anatomical fixation”. The preservation of the aortic bifurcation focuses the columnar blood disrupting forces on the carrefour rather than on the aortic neck minimizing migration risk [6,7]. Different studies have already demonstrated the advantages of this endograft in a relatively small cohort of patients, and its safety and efficacy in short and mid-term follow-up periods [6,10].

***REFERENCES***

1. Volodos NL, Shekhanin VE, Karpovich IP, et al: A self-fixing synthetic blood vessel endoprosthesis. Vestn Khir Im I I Grek 137(11):123–125, 1986. PMID: 3824776
2. Parodi JC, Palmaz JC, Barone HD. Transfemoral intraluminal graft implantation for abdominal aortic aneurysm. Ann Vasc Surg 1991;5:491–499. PMID: 1837729
3. Chuter TA, Donayre C, Wendt G. Bifurcated stent-grafts for endovascular repair of abdominal aortic aneurysm. Preliminary case reports. Surg Endosc 8(7):800–802, 1994. PMID: 7974112
4. Blum U, Voshage G, Lammer J, et al: Endoluminal stent-grafts for infrarenal abdominal aortic aneurysms. N Engl J Med 336(1):13–20, 1997. PMID: 8970935
5. Cao P, Verzini F, Parlani G, et al. Predictive factors and clinical consequences of proximal aortic neck dilatation in 230 patients undergoing abdominal aorta aneurysm repair with self-expandable stent-grafts. J Vasc Surg. 2003 Jun;37(6):1200–5. PMID: 12764265
6. Coppi G, Silingardi R, Tasselli S, et al. Endovascular treatment of abdominal aortic aneurysms with the Powerlink Endograft System: influence of placement on the bifurcation and use of a proximal extension on early and late outcomes. J Vasc Surg. 2008 Oct;48(4):795–801. PMID: 18586447
7. Wang GJ, Carpenter JP. The Powerlink system for endovascular abdominal aortic aneurysm repair: Six-year results. J Vasc Surg. 2008 Sep;48(3):535–545.e3. PMID: 18635335
8. Albertini J-N, Lahlou Z, Magnan P-E, et al, French Powerlink Multicenter Trial Investigators. Endovascular repair of abdominal aortic aneurysms with a unibody stent-graft: 3-year results of the French Powerlink Multicenter Trial. J Endovasc Ther Off J Int Soc Endovasc Spec. 2005 Dec;12(6):629–37. PMID: 16363890
9. Qu L, Raithel D. From clinical trials to clinical practice: 612 cases treated with the Powerlink stent-graft for endovascular repair of AAA. J Cardiovasc Surg (Torino). 2009 Apr;50(2):131–7. PMID: 19329908
10. Diethrich EB. Novel sealing concept in the Endologix AFX unibody stent-graft. J Cardiovasc Surg Torino. 2014;93–102. PMID: 24356051
